# Spiritualism

**Published:** 1856-01

**Authors:** 


*Spiritualism.*— At the recent meeting (1855) of the "Association of Me d ical Superintendents of American Institutions for the Insane," Dr. Luther V. Bell, of the McLean Asylum, (Mass.) presented a paper on what are called "spiritual phenomena," in which we are surprised to notice greater concessions to this modern mania, than we should have expected from a source so eminent in a scientific point of view, as that occupied by Dr. Bell as President of this learned and honored association.

Those of our readers who have been any length of time on our list, are fully aware of the position early taken and steadily maintained by this Journal, viz., that the subject was medically an important one, and deserving of careful study by competent minds. It was in this conviction that our predecessor made his widely-known *exposé* of Mesdames Fox and Fish, in a paper which fixed the charge of fraud and falsehood indelibly upon those disreputable women. And whatever subsequent revelations may prove of some mysterious motive power, transcending all our preconceived ideas, we shall never doubt that its discovery began in a most shameful swindle. We regard it as amply proved that these women made use of simple muscular power and tricks to make money, under the false pretence of communication with the spirit world.

In accordance with this belief in the rascally maternity of spirit rappings, we have ever been disposed to regard all their offspring, in their various forms of manifestation, as referable to two causes—fraud, and hysterical monomania. And when, as has often happened, phenomena have been reported not explicable by this theory, we have been dissatisfied with the evidence on which they rested. The many weak points in the chain of spiritualism, the sickly gabble of its prophetesses, the indisputably second-rate mortality of the most pretentious of spirit literature, bespoke a mundane origin. We had no faith in the latter-day St. Pauls who so lavishly furnished their addenda to Holy Writ. Apropos to this matter of heavenly counsel vouchsafed to this vagabond world, not yet weaned from yellow covered novels at twelve and a-half cents each, Dr. Bell very justly remarks, that

"Whoever reads the very elegant and powerful preliminary treatises of these gentlemen (Judge Edmonds and Dr. Dexter) will not be able to feel that Swedenborg and Lord Bacon, after their nearly one, and more than two centuries' residence amidst the culture and refined senses of the superior spheres, had more than equalled their unpretending amanuenses yet 'in the vale of tears.'"

That is, Dr. Bell estimates the literary ability of Judge Edmonds above that of the distinguished gentlemen in "kingdom come."

Such evidences had reduced our creed of spiritualism to a few points:

1st. The manifestations were unmistakably mortal in their origin.

2d. Very many of them were mere money-making frauds on simple geese born to be plucked, while others were the product of hysterical deceptions.

3d. If any phenomena occurred not to be explained by natural causes so far as known, that some new cause, still natural, would ultimately be discovered for them. And

4th. That as yet no necessity existed for supposing such a hidden cause, the evidence of mystery being wanting.

It is only upon this last point that our views would differ from those of Dr. Bell. Dr. Bell, in the paper read at the meeting referred to, distinctly asserts that a table moved some fifty feet without human contact or machinery, the hands of those "in the circle" being held eighteen inches above it during its progress.

In common with other heretics we have uniformly declared our willingness to discuss spiritualism whenever its statements should come from competent men. Dr. Bell must, when we look at his position and his long training in the phenomena of mind, be considered competent authority, and the whole subject acquires new dignity and importance from the connection of his name with it.

It is satisfactory to know that Dr. Bell is no believer in spiritualism proper. In the moving of tables, the production of raps, and in the correctness of responses to written or verbal questions, he *is* a believer with some important modifications in the latter.

Dr. B. tells what he saw in the way of table-moving, is positive that no deception existed, and that no known mechanical power was applied. In relation to questions, we quote from the American Journal of Insanity:

"Dr. B. then passed to the topic of responses to mental and verbal questions, and gave several narratives of long conversations with what purported to be the spirits of persons dead from twenty to forty years, in which every question he could devise relating to their domestic history, and to events in it known only to them and him, had been truly answered. Some of the questions put mentally—*i. e.* without speaking or writing—had half a dozen correct replies, forbidding, of course, completely, on any doctrine of chances, the contingency of accident or coincidence, as such *mental* questions, *per se*, negative the explanation of previous knowledge on the part of the medium.

"A brief abstract of one of these will give a general idea of their character. Dr. B. had frequently remarked to his "spiritualist" friends, that if any medium could reproduce the essential particulars of a final interview which had occurred between himself and a deceased brother, in 1826, he should be almost compelled to admit that it came from his spirit; because he was sure that he, Dr. B., had never communicated it to any living being. Hence as it had never been known to but two persons, and was of so peculiar, well-marked a character, as not to be capable of being confounded by generalities, he should hardly be able otherwise to explain it. A few weeks afterward, what purported to be the spirit of that brother, narrated the essential particulars of the interview, the place where, down to the well-recollected fact *that he was adjusting the stirrups of his horse*, preparatory to a distant journey, when it was held."

Now here are well attested facts from a competent authority—facts not to be explained on any natural principle within our knowledge. Only yesterday, a very intelligent mechanic told us that last winter his wife was raised bodily a foot from the floor, and suspended without contact, while one might count ten. Our friend had, however, no faith in the spirits; "we had a *great deal of fun* with the manifestations," said he, "but it's all nonsense to attribute it to spirits."

The common mind seems to have jumped at once to the conclusion that anything incomprehensible must be supernatural, and higher intellects have fallen into the same error of logic—for instance, Judge Edmonds.

It is by no means easy, for a mind thoroughly convinced of the rascality of the originators of this moral epidemic, to concede the existence of any phenomena not referable to fraud or self-deception. We are not yet prepared to believe that there is any *new* law of nature developed in this discussion, but for the sake of free debate we will admit it for the nonce, and see whither it ought to lead us.

In the first place it is evident that infinite harm has resulted from the prevailing belief in spiritualism. Aside from the numerous cases of pronounced insanity caused by it, many minds have been unsettled to a degree disastrous to their earthly interests, and often no less fatal to what sane minds still consider the true interests of the soul. All this mischief has resulted from the belief that the occupants of the spirit world, those gone before, who have already solved the enigma of eternity, were permitted to revisit the world of men, and give their counsels for good or evil. This idea, in itself so fruitful in imaginations, so wonderful in our anticipations of its tendencies, so terribly exciting to the feeble minded, is sufficient to account for the harvest of insanities, suicides, and murders which have followed in its track.

Could we once get rid of this notion, could we prove that whatever may be the cause of the manifestations, the spirit world has nothing to do with them, we should rob the subject of half its interest, should reduce it to a mere question of the influence of mind on matter, a study profitable to the philosopher but not attractive to the Miss Nancies who have hitherto been allowed to monopolize the discussion; and in so doing we should at once put an end to all the dangerous results happening to inferior minds.

Such is the tendency of the conclusions at which Dr. Bell has arrived, and looking at the benefit likely to result from his manner of handling the subject, we are not at all inclined to join in the ridicule and censure so lavishly bestowed upon him by some of our cotemporaries, for having given his attention to a subject put under bans.

We again quote from the Journal of Insanity:

"Pretty early, however, in his investigations, Dr. B. began to find that, however correct his spiritual conferees were in most of their responses, the moment a question was put involving a response, the truth of which was unknown to him, uniform failure occurred. Sometimes, where he believed, at the time, that his questions were truly answered, subsequent information had shown him that he had been mistaken. He had answers which he believed to be true, when the facts were decidedly otherwise.

"Pursuing this train of inquiry, he found the 'spirits,' while averring that they could see him distinctly, 'face to face,' never could read the signature to letters taken from an old file, and unfolded *without his having seen the writing.* Yet as soon as he had cast his eye upon the signature, without allowing any one else to see it, it was promptly and correctly reproduced by the alphabetical rappings. And again, when he had made a previous arrangement with his family that they should do certain things every quarter of an hour at home — he, of course, not knowing what — while he was to ask the 'spirit' what was done at the instant, uniform failure occurred. He proved, too, that the theory of the 'spiritualists' to meet such difficulties — viz., that evil or trifling 'spirits' interfered at *their* end of the telegraph — was not tenable. For the responses, just before and after these gross failures, had been eminently and wonderfully accurate, and the 'spirits' not only declared that they saw with perfect clearness what was going on at his house, but denied that there had been any interruption or interference.

"Dr. B. also gave examples where test questions, involving replies *unknown* to the interrogator, had been designedly intermixed with those which were known. The result uniformly was, that the known responses, however curious and far remote, were correctly reproduced, — the unknown were a set of perfectly wild and blundering errors, the response often being obviously formed out of the phraseology of the question, as a *stuck* school-boy guesses out a reply!

"The result of the inquiries of Dr. B. and his friends — for several gentlemen of eminently fitting talents pursued the investigation with him — was briefly this: *that what the questioner knows, the spirits know; what the questioner does not know, the spirits are entirely ignorant of.* In other words, that there are really no superhuman agencies in the matter at all — no connection with another state of existence; but that it bears certain strong analogies to some of the experiences of *clairvoyance*, in that mysterious science of animal magnetism, as it has been protruding and receding for the last hundred years."

We admire the bold spirit in which Dr. Bell has handled this subject, and willingly endorse the sentiment of his concluding remarks:

"Dr. B. concluded by the expression of his full conviction that, while the faith in spirits must be given up, as connected with these facts, it was a topic, whether regarded as a psychical novelty or even as a delusion, cutting deeply into the very religious nature of our people, which was worth our fullest examination. *There were great, novel, interesting facts here.* They had not been treated fairly and respectfully, as they should have been. The effect was, that the community, knowing that here were *facts*, if human senses could be trusted at all, went away from those who should have thrown light upon the mysteries, but who would not or could not, to those who gave some explanation, even if it was one which uprooted all previous forms of religious faith. He hoped that the members of this association, who were as much required to examine this topic as any order of men, except, perhaps, the clergy, would not be afraid of looking it in the face, from any apprehension of ridicule, or of degrading their dignity."

---

We give place, with pleasure, (at the request of a friend who knows Dr. Benedict well) to the following commendatory notice:

From the Medical Examiner, September, 1855.

"There is no class of invalids who require the comforts, conveniences, and even the luxuries of life more than those threatened with consumption; and yet it oftentimes has happened to those who have sought the equable temperature of Florida during the winter and spring months, to be so seriously inconvenienced for the want of the proper accommodation, adapted to their condition, that the benefits they had hoped to derive by wintering in the south were much diminished.

"We are glad to learn that an establishment will soon go into operation to which those suffering from such affections may resort with every confidence, both in regard to its hygienic management and the medical skill of its attending physician.

"This *Sanitarium* for affections of the throat and lungs, is to be under the care of Dr. N. D. Benedict, late superintendent of the New York State Lunatic Asylum.

"It is located at Magnolia, East Florida, on the river St. Johns, between Jacksonville and St. Augustine, at a distance of about twenty miles from the seaboard. It is one day's journey, by steamboat, from Savannah and Charleston, and four days, by steamer, from New York and Philadelphia, via Charleston or Savannah.

"The climate of East Florida is probably better adapted, as a winter residence, for invalids with delicate lungs, than any other part of the United States. The mean temperature of the winter months is about 60°; frost is rarely seen, and the little rain that falls is rapidly absorbed by a dry, sandy soil, shaded by pine forests.

"After much observation and deliberation, the above site was chosen, believing it to possess as many, if not more, advantages than any other location in the country.

"The house, which is commodious, having large airy chambers, and in every respect well constructed for the purpose, will be opened in November next for the reception of invalids, who may or may not be accompanied by their friends.

"We have known Dr. Benedict for many years, and can bear warm testimony, from personal experience, to his skill as a physician and his warm-heartedness as a man. More particular information may be obtained by addressing him at Philadelphia."

---

We have heretofore omitted to mention the appointment of Dr. B. H. Lemon to the place of House Physician to the Buffalo Hospital of the Sisters of Charity. Our accidental delay enables us to say, that during the three months of his residence, thus far, Dr. Lemon has evinced a large degree of fitness for the place, and of ready skill in the treatment of disease.

